# Transcriptional Reprogramming in Rumen Epithelium during the Developmental Transition of Pre-Ruminant to the Ruminant in Cattle

**DOI:** 10.3390/ani11102870

**Published:** 2021-09-30

**Authors:** Ransom L. Baldwin VI, Mei Liu, Erin E. Connor, Timothy G. Ramsay, George E. Liu, Cong-Jun Li

**Affiliations:** 1 Animal Genomics and Improvement Laboratory, Agricultural Research Service, Beltsville, MD 20705, USA; ransom.baldwin@usda.gov (R.L.B.VI); Liumei_1989@163.com (M.L.); eeconnor@udel.edu (E.E.C.); George.liu@usda.gov (G.E.L.); 2 Animal Biosciences and Biotechnology Laboratory, Agricultural Research Service, Beltsville, MD 20705, USA; timothy.ramsay@usda.gov

**Keywords:** gene expression, nutrition-gene interaction, rumen development, rumen epithelium, transcriptome

## Abstract

**Simple Summary:**

The rumen is the critical organ mediating nutrient uptake and use in cattle. Health development is essential to ensure animal feed efficiency. In this report, we present an analysis of gene expression dynamic in rumen epithelium during the transition from pre-ruminant to ruminant in cattle fed with hay or concentrated diets at weaning. The global shifts in gene expression and correlated transcription factors activities indicate transcriptional reprogramming during weaning. Transcriptional reprogramming in rumen epithelial tissue reflects critical nutrient-gene interactions occurring during the developmental progression. The results unveiled that nutrient-gene interactions compel transcriptional reprogramming. Our findings also suggest that this transcriptional reprogramming is the molecular basis of the transitional development of pre-ruminant to the ruminant in cattle.

**Abstract:**

We present an analysis of transcriptomic dynamics in rumen epithelium of 18 Holstein calves during the transition from pre-rumination to rumination in cattle-fed hay or concentrated diets at weaning. Three calves each were euthanized at 14 and 42 d of age to exemplify preweaning, and six calves each were provided diets of either milk replacer and grass hay or calf starter to introduce weaning. The two distinct phases of rumen development and function in cattle are tightly regulated by a series of signaling events and clusters of effectors on critical pathways. The dietary shift from liquid to solid feeds prompted the shifting of gene activity. The number of differentially expressed genes increased significantly after weaning. Bioinformatic analysis revealed gene activity shifts underline the functional transitions in the ruminal epithelium and signify the transcriptomic reprogramming. Gene ontogeny (GO) term enrichment shows extensively activated biological functions of differentially expressed genes in the ruminal epithelium after weaning were predominant metabolic functions. The transcriptomic reprogramming signifies a correlation between gene activity and changes in metabolism and energy production in the rumen epithelium, which occur at weaning when transitioning from glucose use to VFA use by epithelium during the weaning.

## 1. Introduction

Weaning is the process of gradually introducing an infant human or another neonatal mammal to its adult diet while withdrawing its mother’s milk supply. The forestomach compartments of ruminants are underdeveloped at birth. They are virtually non-functioning, making neonatal ruminant digestion like that of monogastric mammals. To facilitate the digestion of grasses and complex carbohydrates characteristic of the mature ruminant diet, the rumen, reticulum, and omasum must increase in size and capacity to house the symbiotic microbiota until the development of these organs is completed after weaning [1]. Besides, rumen papillae develop and grow in length and breadth, greatly enhancing the rumen surface area to absorb nutrients [2,3]. In both growing and lactating ruminants, the rumen microbes digest simple and complex carbohydrates (fiber), for which the animals lack digestive enzymes to convert them into short-chain fatty acids (VFAs). These VFAs consist mainly of acetic, propionic, and butyric acids, which the animal can absorb and use as energy sources. VFAs can provide 50% to 70% of the energy requirements of cattle and other ruminants [4]. There is only a minimal level of fermentation occurring in the rumen of pre-ruminants. They are mainly dependent on the lower GIT digestion for energy and nutrient requirement rather than rumen fermentation. The dramatic change that must occur before weaning is exemplified by the rumen compartment, which increases from 30% of gut capacity to 70% post-weaning [5]. This plasticity also occurs in other physiological states, such as lactation, where increases in mass as a percentage of empty body weight (body weight minus gut contents) are observed in adult cows [6,7]. In contrast to monogastric animals, the forestomach compartments of ruminants have highly specialized structures and functions, leading to substantial differences in digestion and physiology between ruminants versus pre-ruminants or monogastric animals. 

Rumen development is an essential factor regulating early solid feed intake and fundamentally affects on cattle’s growth performance and feed efficiency. Diet composition represents one of the most critical factors affecting the establishment of a viable rumen microbiota and, subsequently, fermentation, rumen development, and host production performance [8,9]. In the pre-ruminant calf, liquid feed, often in the form of a milk replacer, typically bypasses the rumen compartment via closure of the esophageal groove and directly enters the abomasum for acid digestion. However, closure is not always effective; thus, normal leakage of milk straight into the rumen plays a vital role in its development by providing ample nutrients and suitable habitat for microbial settlement. Rumen colonization by microbes begins during the birthing process. These microbes multiply and become highly active and ready to ferment solid feed from one week of age [6]. Intriguingly, the VFAs produced by the early microbiota are associated with triggering the physical development of the epithelium and regulating the differentiation of rumen tissue metabolism from glucose to VFAs and ketogenesis [1,10]. Thus, establishing the rumen microbiota of pre-ruminant calves lays a solid foundation for the transition from the pre-ruminant to the ruminant state, a critical stage of digestive adaptation in cattle [11]. 

Transcriptional reprogramming refers to the phenomenon of global shifts in gene expression typically initiated by transcription factors. During transcriptional reprogramming, the expression of specific genes is elevated, whereas other genes are repressed, compared to the previous state [12]. Reprogramming of transcription is required to maintain homeostasis under adverse growth conditions [13,14]. Although the study of rumen development has attracted much attention [2], a comprehensive understanding of the genomic activities controlling ruminal morphological and physiological changes occurring during this critical time in calf development remains incompletely defined. 

The downstream gene expression activities activated by developmental transition remain largely unknown. Particularly, system-wide analysis of the underlying dynamics of the transcriptome changes is lacking. We reported earlier using microarray to identify genes differentially expressed between the pre-ruminant and ruminant stages of differentiation induced by diet. The objects of the original study were to identify genes and gene networks contributing to rumen growth and development targeted the mechanisms regulating these processes to improve weaning strategies for young calves [15]. However, the next-generation sequencing (NGS) technology gives a complete categorization of the transcriptome. The objectives of the present study are to use RNA-sequencing combined with bioinformatics approaches to characterize the dynamics of the rumen epithelial transcriptome of dairy calves in the developmental transition of pre-ruminant (before weaning, BW) to the ruminant after weaning (AW); to apprehend the genomic activities triggering rumen development; and to understand the molecular basis and mechanisms underlying the rumen development. Our results identified the global shifts of transcriptional activities in the ruminal epithelial tissue associated with physical and metabolic development. Upstream regulator and transcription factor (TF) families displayed a distinctive pattern during the shift in developmental states, signifying transcriptional reprogramming. Transcriptional reprogramming in rumen epithelial tissue indicates critical nutrient-gene interactions occurring during the developmental progression.

## 2. Materials and Methods

### 2.1. Animals and Tissue Collection

**Figure 1:**
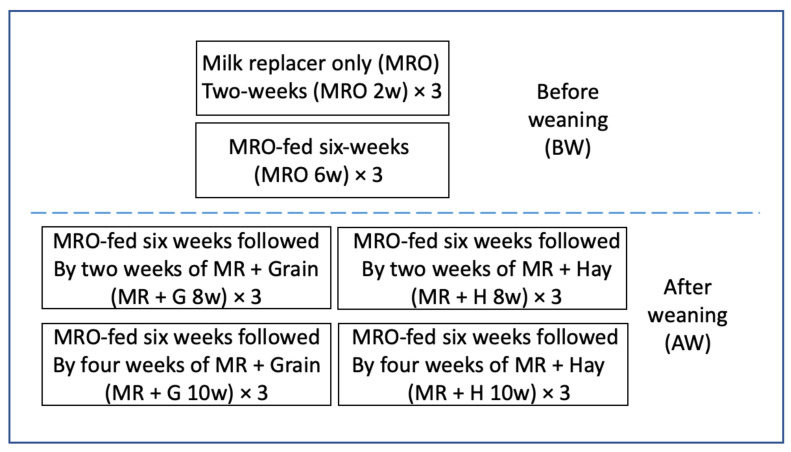
Diagram describing feeding scheme of the 6 developmental groups evaluated. 18 calves were divided among 6 groups of 3 calves each (6 × 3). Groups above the dashed horizontal line represent those preweaning, and groups below the dished line represent those post-weaning.

Animals and tissue collection were described in our previous report [15]. Briefly, eighteen Holstein bull calves averaging 1.3 days of age and 89 kg bodyweight purchased from a private dairy farm in New Windsor, MD, USA, were used in the study. Calves were housed individually in 7.3-m^2^ concrete pens with a rubber mat as a bedding surface with no bedding material to avoid confounding dietary treatments if consumed by calves. As illustrated in Figure 1, calves were assigned randomly to one of three dietary treatments (six calves/treatment): milk replacer only (MRO), milk replacer + grain-based commercial calf starter (MR + G), or milk replacer + orchard grass hay (MRO + H) fed free choice. Three calves each were euthanized at 14, and 42 d of age for tissue collection to exemplify preweaning, and six calves were provided diets of either milk replacer + orchard grass hay. Six calves were provided milk replacer + calf starter to set off weaning and development of rumen papillae. At 56 and 70 d of age, three calves from the MH and MG groups were euthanized for the collection of rumen epithelium. Calves were provided 0.23 to 0.45 kg of calf starter daily, depending on body weight. All calves had continuous access to water and were fed twice daily with the commercial milk replacer for six weeks (1.9 L per feeding for a total of 3.8 L/day as recommended by the manufacturer). Calves were bottle-fed and transitioned to bucket feeding by six days of age, then transitioned to either MR + H or MR + G diets wherein milk replacer was still provided twice daily (3.8 L total) in addition to hay or calf starter. Consumption of hay was monitored to ensure consumption by calves and maintenance of ad libitum access. The quantity was increased by 0.23 kg once calves consumed all fed grain for three consecutive days. All calves were euthanized by captive bolt followed by exsanguination. Calves in the MRO group were euthanized at two weeks of milk replacer only feeding (MRO 2w; *n* = 3) and at six weeks of milk replacer only feeding (MRO 6w; *n* = 3) to represent two stages of pre-ruminant development. Among the MR + H and MR + G groups, calves were euthanized after 2 weeks (*n* = 3 per diet) or 4 weeks (*n* = 3 per diet) of access to solid feed to represent development at two stages of weaning on either a grain-based (MR + G 8w and MR + G 10w) or hay-based (MR + H 8w and MR + H 10w) diet [16]. 

The rumen tissue was taken from the ventral sac below the cranial pillar, which in a ruminant is consistently exposed to rumen liquor and sometimes the mat during contractions. There are distinct visual cues present to ensure the tissue was predominately exposed to rumen liquor prior to slaughter. The collected rumen tissues were rinsed with tap water, followed by rinsing in ice-cold physiological saline. According to manufacturer instructions, subsamples (~600 mg) of epithelial tissue were fixed in RNAlater (Life Technologies, Grand Island, NY, USA) RNA stabilization solution according to manufacturer instructions and stored at −80 °C until RNA extraction. 

### 2.2. RNA Sequencing and Gene Expression Analysis

The RNA extraction procedure was reported previously [17]. Briefly, total RNA was extracted using Trizol (Invitrogen, Carlsbad, CA, USA) followed by DNase digestion and Qiagen RNeasy column purification (Qiagen, Valencia, CA, USA). The RNA integrity was verified using an Agilent Bioanalyzer 2100 (Agilent, Palo Alto, CA, USA).

After quality control procedures, RNA was sequenced at 150-bp/paired-end sequence reads using RNA-sequencing service supplied by Novogene Corporation, Inc. (UC Davis Sequencing Center, Davis, CA, USA). The CLC Genomics Workbench (v20; Qiagen Bioinformatics, Redwood City, CA, USA) was used for further RNA-Seq data analysis. Trimmed reads were aligned to the newest bovine reference genome assembly (ARS-UCD1.2) [18]. Gene expression levels of mapped reads were normalized as reads per kilobase of exon model per million mapped reads (RPKM) using the CLC transcriptomic analysis tool. To ensure the accuracy of estimated RPKM values and remove the auxiliary data, only genes with RPKM > 1 in at least one sample were analyzed. Expression levels of each gene in all samples were log2 converted in the following analysis. Principal component analysis (PCA), heatmap, differentially expressed genes (DEGs), and gene ontology (GO) analysis of DEGs were all performed using CLC Genomics Workbench. The enrichment of specific GO terms was determined based on the Fisher exact test. The DEGs were defined only if the corresponding *p*-value of false discovery rate (FDR) were less than 0.05. Thus, genes that contributed most to separate different cell groups were determined. Venn diagram was performed using jvenn, an interactive Venn diagram viewer [19].

CLC Genome workbench (Qiagen) was also used for Principal Component Analysis (PCA). PCA was used to finds patterns about whether the samples come from different treatment groups or have phenotypic differences in this study. PCA was performed using all expressed genes. PCA simplifies the complexity in high-dimensional data while retaining trends and patterns. It does this by transforming the data into fewer dimensions, which act as summaries of features. High-dimensional data are very common in biology and arise when multiple features, such as the expression of many genes, are measured for each sample [20]. The goal of PCA is to identify directions (or principal components) along which the variation in the data is maximal. As described before [21], Ingenuity Pathway Analysis (IPA, Qiagen Bioinformatics, Germantown, MD 20874, USA) was used to analyze gene expression datasets. The analysis of canonical pathways identified the pathways from the IPA library of canonical pathways that were most significantly represented in the data set. The set of gene identifiers and corresponding expression values were uploaded into the IPA application. Each gene identifier was mapped to its corresponding gene object in the Ingenuity Pathways Knowledge Base. These genes, called focus genes, were overlaid onto a global molecular network developed from information in the Ingenuity Pathways Knowledge Base. Networks of these focus genes were then algorithmically generated based on their connectivity in the IPA database. IPA also was used for comparison analysis. The datasets of identified DEGs were used in the comparison analysis. The z-score is a statistical measure of how closely the actual expression (or other measurement types) pattern of molecules in the dataset compares to the pattern that is expected based on the literature for a particular annotation and was calculated in IPA as described in the IPA publication [22]. 

## 3. Results

### 3.1. RNA Sequencing and Transcriptomic Profiling of Rumen Epithelial Tissue

RNA sequencing statistics are listed in Table 1. A total of 21,648 genes were annotated from all individual RNA sample sequencing. The individual gene expression values were used to compare DEGs among groups.

### 3.2. Impact of Weaning Transition on Transcriptome Atlas Dynamics in Rumen Epithelium

**Figure 2:**
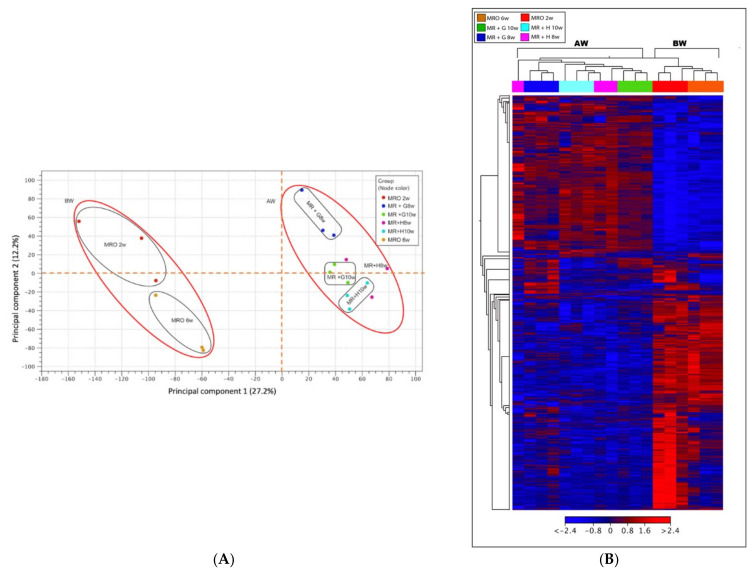
Gene expression dynamics are weaning state-specific. (**A**) PCA comparing gene expression patterns of the 6 groups defined in Figure 1. Each dot denotes a single biological replicate, and the ellipses represent the groups. The PCA (PERMANOVA tests) distinguishes temporal patterns where the transcriptomes cluster separately between groups pre- and post-weaning. (**B**) The hierarchical heatmap (Euclidean distance) of selected DEGs of all 6 groups. The heatmap shows the average linkage and shift of DEGs between BW and AW groups, and the consistency within the pre-weaning and post-weaning groups. The rows of the matrix are individual genes, and the columns correspond to each sample. The heat map is scaled with red and blue colors proportionate to gene expression value (red represents up-regulated and blue represents down-regulated, color range: log_2_ fold change). AW: after weaning; BW: before weaning.

The transcriptome atlas provides the number of transcripts expressed for individual genes before and after weaning and the quantity of transcript expression for individual feeding schemes (i.e., grain or hay in diet). We estimated overall weaning, stage-specific diversity by comparing mRNA populations using PCA and a hierarchical heatmap (Figure 2A,B, respectively). The PCA distinguished the temporal expression patterns between groups before- and after-weaning (BW and AW) with samples from weaned calf rumen (MR + G 8w and MR + H 8w; MR + G 10w and MR + H 10w) forming different subgroups that clustered separately from the two BW groups (MRO 2w and MRO 6w). This analysis illustrates that each group-derived transcriptome is reproducible, as the biological replicates are mostly clustered together, and differential groupings of transcriptomes among the BW and AW stages were distinct. Moreover, sub-groups were generally different for each feeding scheme at weaning with MR + H 8w group overlap with the MR + H 10w and MR + G 10w groups. We also see a very interesting trend from the PCA analysis diagram that the variation among individual replicates diminished after weaning. A common method of visualizing gene expression data is to display it as a heatmap. The heatmap combined with the clustering method groups samples together based on their gene expression pattern similarity. As shown in Figure 2B, hierarchical clustering is consistent with the PCA results as the disparity in gene expression between BW and AW groups. 

**Figure 3:**
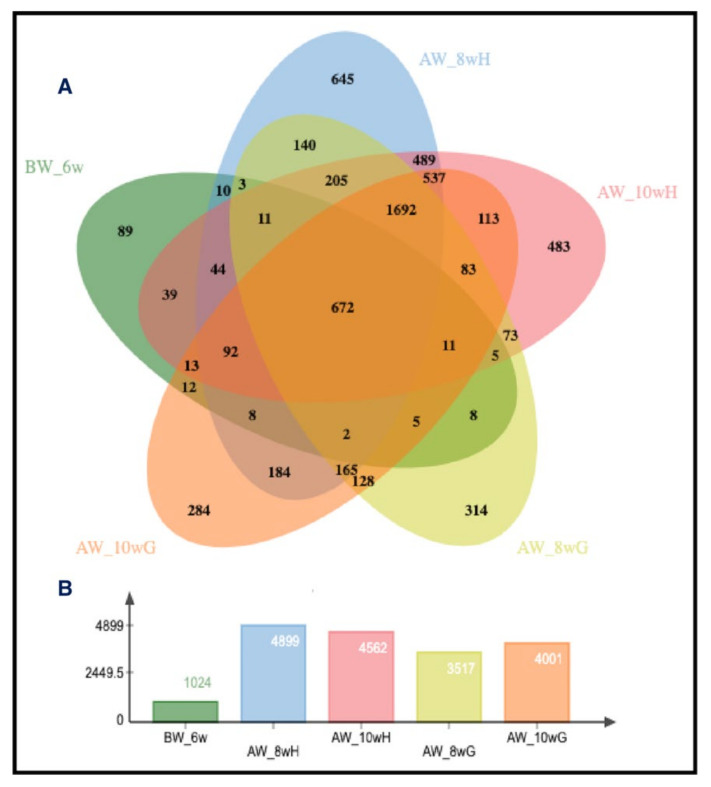
(**A**) A five-way jvenn Diagram (adapted from [19]) illustrates the number of DEGs for each group comparison, as well as the unique and overlapping DEGs among all of the groups. There are 672 DEGs common in all five groups. Moreover, the diagram also shows the number of unique DEGs in each of the groups. In the BW6w group, there are only 89 unique DEGs, compared to 648 DEGs in AW 8wH, 483 DEGs in AW 10wH, 314 DEGs in AW 8wG, and 284 DEGs in AW 10wG. (**B**) The number of DEG represents each group compared to the two-week-old MRO group.

We then compared the transcript expression changes after weaning against the rumen epithelial tissue’s transcriptome from the before-weaned calf group fed milk replacer only for two weeks. From a total of 21,648 annotated genes identified, those DEGs expressed in ruminal epithelial tissue from each developmental stage and diet are presented in a jvenn diagram (Figure 3A) [19]. For context, while there were only 1024 DEGs identified between rumen epithelium of the two pre-weaning stages (MRO 2w versus MRO 6w), there were 4899 DEGs identified from rumen epithelium of calves in the MRO 2w group compared to the MR + H group at week 8 of the study (MR + H 8w; 2 weeks post-weaning fed hay) and 3517 DEGs between MRO 2w with the MR + G group at week 8 (Figure 3A). Likewise, for the epithelial tissues collected at week 10 of the study, those from calves fed hay for 6 weeks (MR + H 10w) had 4562 DEGs compared to the MRO 2w group, whereas calves fed grain for 6 weeks (MR + G 10 w) had 4,001 DEGs. These changes in the number of DEGs at each age group and feeding scheme versus the MRO 2w group are presented graphically in Figure 3B. These analyses further illustrate the dominant impact of the developmental state on the transcriptome. 

### 3.3. Transcriptional Reprogramming Induced by Weaning in Ruminal Epithelial Tissue and Reveals Significant Changes in Cellular Metabolism-Related Gene Regulation

As compared to our previous study with microarray [15], more DEGs were discovered by RNA-sequencing. More significantly, by an integrative bioinformatics approach, we revealed the dynamics of the rumen epithelial transcriptome of dairy calves in the development transition of pre-ruminant to ruminant. To explore how the transcriptome varied over time and during rumen development in response to feeding scheme (i.e., hay versus grain), the DEGs across groups were identified, and an ANOVA-like differential expression (ALDEx, for identification of genes with greater between- to within-condition differences procedure embedding in the CLC genomics workbench 20 software, Qiagen, Bioinformatics, Germantwon, MD 20874) was used to identify DEGs with more significant between-condition differences than within-condition differences [23]. We then investigated gene ontogeny (GO) term enrichment, which occurred between pre-rumination to post-rumination in rumen epithelium using the transcriptomes from each of the six calf groups. The GO elucidates our knowledge of the biological domain with characteristics of molecular function, molecular activities performed by gene products, and molecular function terms illustrate activities that occur at the molecular level. The GO-term enrichment analysis reflected transitions in functional (physical and metabolic) development of ruminal epithelium and revealed significant cellular metabolism-related gene regulation.

**Figure 4:**
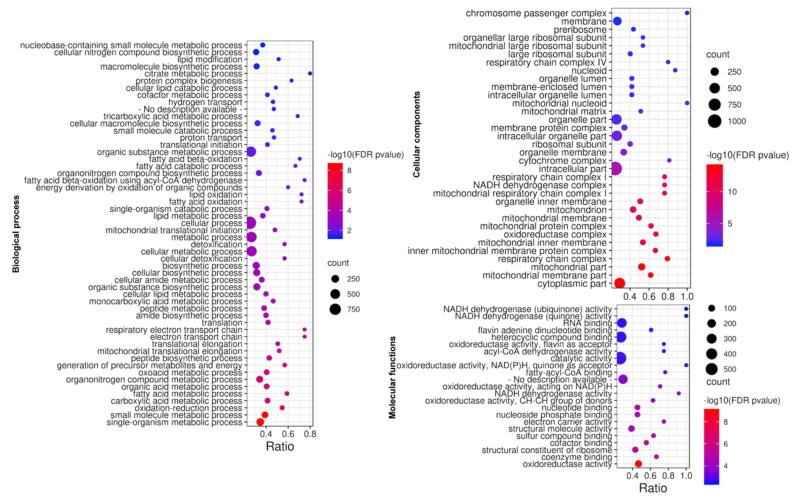
GO enrichment bubble plot is used for GO pathway enrichment analysis. The bubble plot displays the most significant terms after performing GO enrichment analysis. Three plots represent Gene ontology (GO) term enrichment in biological processes, molecular functions, and cellular components, respectively. Bubble colors represent the *p*-value of False Discovery Rate (FDR pvalue). Bubble sizes indicate the number of differentially expressed genes (count). Furthermore, the ratio (Number of differentially expressed genes/number of detected genes) was presented on the X-axis, and the GO pathway terms were displayed on the Y-axis.

The significant GO terms with subontology (the biological processing, molecular functions, and cellular components, with FDR *p*-value < 0.05) (Figure 4). The GO terms analysis revealed that the most DEGs were enriched in biological processes related to energy metabolisms, such as cellular metabolic processes and cellular lipid catabolic processes. The most significant molecular functions impacted by weaning included ‘oxidoreductase activity,’ ‘coenzyme binding,’ and ‘structural constituent of ribosome.’ Lastly, enrichment for cellular components was most significant for the ‘mitochondrial part,’ further reflecting stark changes in metabolism and energy production in rumen epithelium, which must occur at weaning when transitioning from glucose use to VFA use by epithelium during the weaning process. 

All related GO terms were enriched after weaning, indicating transcriptional reprogramming of rumen epithelial tissue induced by the weaning process. As indicated by GO terms, significantly activated biological functions in the ruminal epithelium were predominant metabolic functions. The results indicate critical nutrition-gene interactions occurring during the dietary shift from liquid to solid feeds, which prompted transcriptional reprogramming in rumen epithelial tissue during the developmental progression of ruminant digestion.

### 3.4. The Developmental Shift of Gene Expression among the Temporal Groups during the Weaning Transition

**Figure 5:**
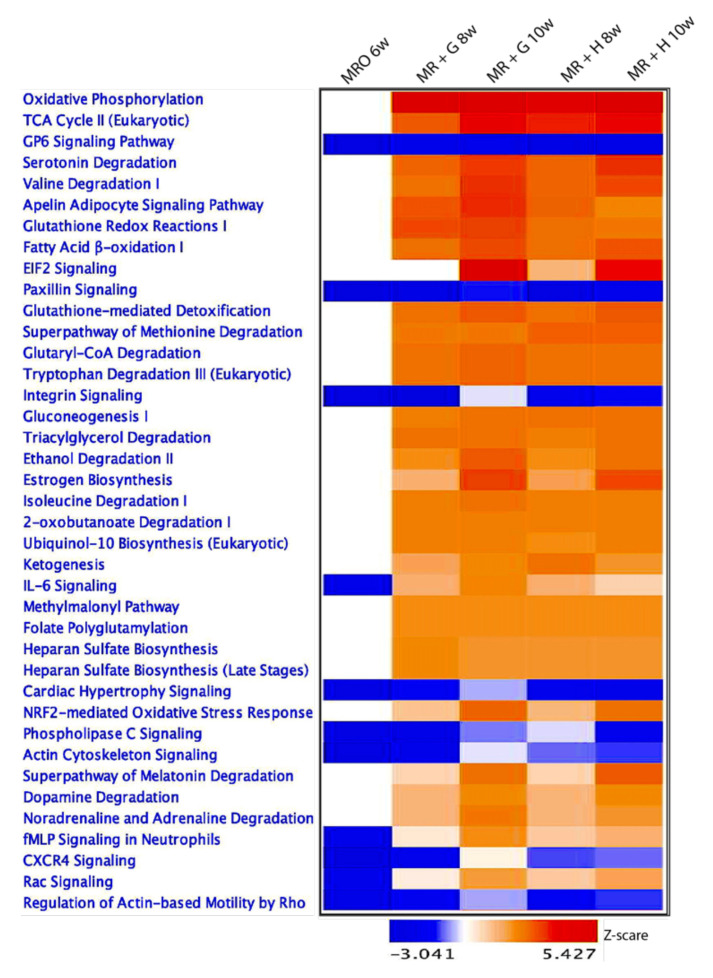
Comparison Analysis-Canonical pathways: Canonical pathway analysis reveals transcription reprogramming with clusters of the critical pathways to control the patterns of ruminal functions during the transition of pre-rumination to rumination in rumen epithelium. Heatmap shows comparisons of individual temporal groups and feeding schemes (MRO 6w, MR + H 8w, MR + H 10w, MR + G 8w, and MR + G 10w) with the MRO 2w groups. The transcriptomes were first analyzed with IPA to identify the significant canonical pathways, and then a comparison was performed between individual temporal groups to the MRO 2w, respectively. The transcriptome atlas of the preweaning groups (i.e., MRO 6w versus MRO 2w) revealed no significant up-or down-regulated canonical pathways. However, many canonical pathways, especially those related to metabolism, were activated after the 2 to 4 weeks of supplemented solid feeding in the form of hay or grain.

**Figure 6:**
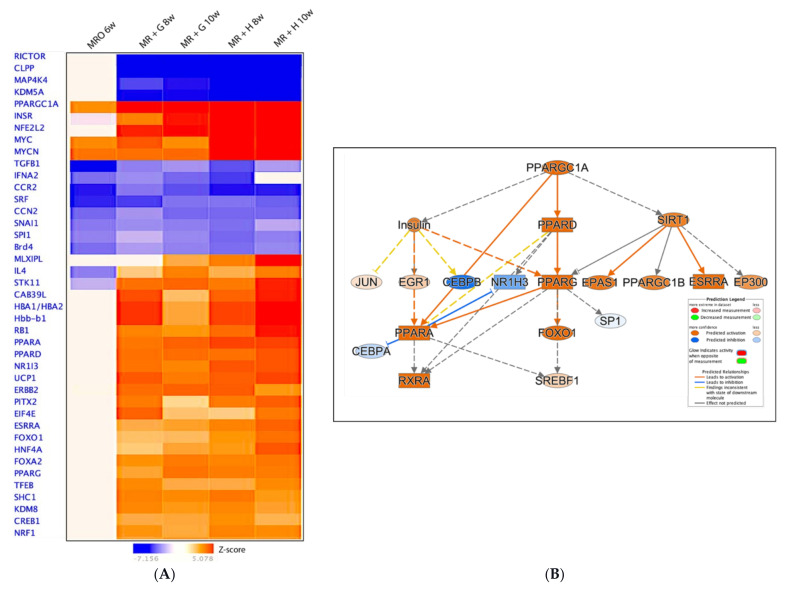
(**A**) Comparison analysis-the upstream regulators: Clusters of effectors on the key pathways to control the patterns of ruminal functions. Upstream regulator analysis showed activation of many upstream regulators and transcription factors during the transition of weaning. (**B**) Predicted mechanistic network of the upstream regulator PPARGC1A: The mechanistic network contains 19 regulators in the dataset (and 462 downstream genes, See Supplemental Table S1). The entries in Supplemental Table S1 show how a target (row) supports the prediction of a regulator (in each column) in the PPARGC1A mechanistic network.

From comparisons of individual temporal groups and feeding schemes (MRO 6w, MR + H 8w, MR + H 10w, MR + G 8w, and MR + G 10w) with the MRO 2w group, we used datasets of identified DEGs to separate the likely affected canonical pathways, cellular functions, upstream regulators, and transcription factor (TF) families regulating the observed temporal gene expression patterns both unique and shared among gene expression patterns during the weaning transition. The putative impact of this developmental shift of gene expression also was realized in the canonical pathway analysis and upstream regulator analysis (Figure 5 and Figure 6). Canonical pathway analysis revealed transcriptional reprogramming in rumen epithelium identifying clusters of critical pathways that likely regulate ruminal function changes (physical and metabolism-related) during the transition from pre-rumination to rumination. Comparing the transcriptome atlas of the preweaning groups (i.e., MRO 6w versus MRO 2w) revealed no significant up- or down-regulated canonical pathways. However, many canonical pathways, especially those related to metabolism, were activated after the 2 to 4 weeks of supplemented solid feeding in the form of hay or grain (Figure 5). The most activated pathway, ‘oxidative phosphorylation,’ is the metabolic pathway in which cells use enzymes to oxidize nutrients, releasing the chemical energy of molecular oxygen used to produce adenosine triphosphate (ATP) [24]. The oxidative phosphorylation pathways occur by enzyme systems in the mitochondria, like other metabolic pathways such as the ‘TCA cycle’ and ‘fatty acid β-oxidation.’ Canonical pathways involved in both the TCA cycle and fatty acid β-oxidation were upregulated during the transition reflecting the change from glucose to VFA. 

Transcriptional reprogramming is typically initiated by transcription factors [12]. Our results indicated that upstream regulator and transcription factor (TF) families displayed a distinctive pattern during the developmental state shift. The most dramatic (up- or -down- Z-score) TF expression changes during the weaning transition are shown in Figure 6A. Notably, activation Z-scores of five known upstream regulators (PPARGC1A, INSR, NFE2L2, MYC, MYCN, and PPARA) were higher in the hay-fed groups (Figure 6A). To elucidate the predicated effects of upstream regulators on the reprogramming of gene activities, we summarized one of the mechanistic networks, PPARGC1A, in MR +H 10W group compared to MRO 2w (Figure 6B). The upstream regulator PPARGC1A controls energy, and nutrient homeostasis plays an essential role in metabolic reprogramming in response to dietary availability [25]. PPAGC1A regulates the activities of multiple downstream genes impacted within our dataset *(p* = 1.12 × 10^−29^ of overlap). The mechanistic network contains 462 downstream genes and 19 regulators in the dataset (Supplemental Table S1). The level of downregulation of these transcription factors except TGFB1 and SRF was most significant in the calves fed hay post-weaning. Because the patterns of change for the different feed groups observed at the canonical pathway-level were not noticeably affected (Figure 5), upstream regulators were likewise minimally affected between the feeding schemes (MR + G versus MR + H). 

**Figure 7:**
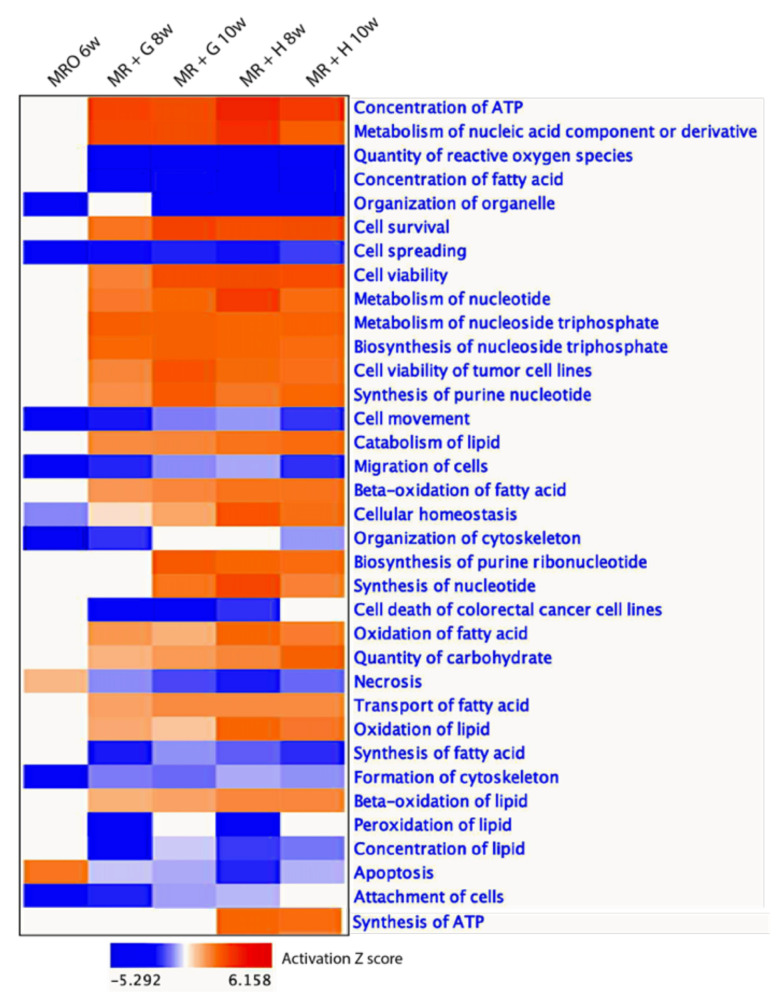
Comparison analysis-cellular function: Functional analysis of differentially expressed genes during the transition from pre-rumination to rumination in rumen epithelium.

A comparison of cellular functions affected by weaning shows the functional shift induced by transcriptome reprogramming during the weaning transition (Figure 7). Many of the functions, especially those related to metabolic functions, were consistently activated after the 2 to 4 weeks of supplemented solid feeding in the form of hay or grain. Other activated functions included ‘transport of fatty acids,’ ‘cell survival,’ ‘cell viability,’ and ‘concentration of ATP.’ Some functions were deactivated, such as ‘quantity of reactive oxygen species,’ ‘concentration of fatty acid,’ ‘synthesis of fatty acid,’ ‘concentration of lipid,’ ‘apoptosis,’ and ‘necrosis.’ 

## 4. Discussion

Here, we use RNA-sequencing combined with an integrative bioinformatics approach to characterize the dynamics of the rumen epithelial transcriptome of dairy calves in the developmental transition of pre-ruminant to the ruminant after weaning. The bioinformatic analysis illustrated the global shifts of transcriptional activities in the ruminal epithelial tissue. Our results designate the two distinct phases of rumen transcription activities in cattle before and after weaning. The global shifts in gene expression and correlated TF activities indicate transcriptional reprogramming during weaning. The results unveiled that nutrient-gene interactions compel transcriptional reprogramming. Our findings also suggest that this transcriptional reprogramming is the molecular basis of the transitional development of pre-ruminant to the ruminant in cattle.

The transcriptome is known to have distinct profiles unique to cell type, developmental stage, health status, and biological function [26]. The shifts in gene activities observed in the present study reflect the transcriptomic reprogramming required to induce developmental and functional changes in the rumen epithelium during cattle weaning. The animal transitions from one functioning essentially as a pseudo-monogastric (pre-rumination) to a fully functional ruminant. These two distinct phases of cattle rumen’s development and function are tightly regulated by a series of signaling events and clusters of effectors on critical pathways. Our analysis helps to identify these signaling events and effectors. 

The rumen epithelial lining has several distinct characteristics, making it an excellent model for studying nutrient-gene interactions occurring in vivo under naturally occurring developmental processes critical in ruminant production settings. Proper development and function of the rumen at weaning are crucial to the health and productivity of dairy calves. Research efforts on the development of rumen and feeding methods mostly focus on the physical development of rumen [7,27]. Our earlier report also showed that dietary change induces the morphological changes of the rumen and gross rumen papillary development, and overall, visible differences in rumen mucosal development and morphology were accomplished by feeding the MRO, MG, and MH diets [15]. In the current analysis, a total of 1024 genes were differentially expressed between two stages of rumen development before weaning (MRO 2w and MRO 6), indicating a non-feeding-induced component to development. More dramatic, however, is the observed change in gene expression in ruminal epithelium among the post-weaning groups. The shifts in genes expressed by transcript profiling in the present study reflect the complex reprogramming required to induce and maintain new developmental and functional changes necessary for the rumen epithelium to transition into an efficient organ suitable for the mature ruminant diet. The very specific rumen epithelial transcriptome reflects the morphological and functional differences of rumens during weaning transition. Elucidating this organ’s transcriptomic dynamics, especially related to intermediary substrate metabolism, is essential to better interpret findings from in vivo and in vitro studies of rumen development. 

In practice, during weaning, young ruminants are transitioned away from a liquid diet of milk or milk replacer onto a solid feedstuff (e.g., grain, grass, or hay). The transition profoundly impacts transcriptome atlas present in the rumen epithelium as the developmental status dictates differential function. The change in expression of metabolism-related genes is positively correlated with dry matter intake, indicating that the enhanced metabolic process may promote rumen development during weaning [28]. Integrative analysis of transcriptomes suggested an overall increase in carbohydrate and fatty acid-specific metabolism, such as changes in the cellular metabolic pathways after weaning, as consistent with previous reports [15,29,30,31,32].

The transcription factor E2F1 modulates the expression of specific chromatin components in oligodendrocyte progenitor cells during the transition from proliferation to differentiation. Specifically, E2F1 targets cell cycle genes and chromatin components, including those modulating DNA methylation [33]. In our study, the E2F1 gene expression was downregulated in the rumen tissue from weaned groups, mostly consistent with our previous results using cultured primary rumen epithelial cells treated with butyrate. Thus, E2F1 is a putative upstream regulator for both proliferation and development in the ruminal epithelium. Likewise, TGFB1 is also a negative regulator of proliferation and is a known inducer of apoptosis beyond its fibrogenic effects, leading to *trans* differentiation of hepatic stellate cells into myofibroblasts [34]. Like the findings of E2F1, TGFB1 is significantly downregulated in rumen epithelial tissues from the weaning groups. Down-regulation of both E2F1 and TGFB1 in the rumen epithelium from weaning calves is consistent with our previous in vitro experiment report of primary rumen epithelial cells treated with butyrate [35]. 

The transcriptome defines ruminal development status and regulates the transition necessary for ruminal development. We observed reprogramming of the transcriptome in the present study, resulting in changes in gene expression, canonical pathways, and cellular functions. Besides, upstream regulators, majorly transcription factors, were demonstrably enriched or activated post-weaning. We can assume that the maturation of rumen epithelium is mediated by transcriptional reprogramming in response to establishing a viable microbiota and its generation of VFAs, likely butyrate [36]. 

Understanding the processes at a transcriptional reprogramming level will undoubtedly help us understand how cellular metabolism changes related to gene regulation. The gene expression profile of the rumen epithelium has been used to understand molecular changes during weaning [31,37] or in dietary transitions of adult cattle [37,38]. The establishment of rumen fermentation and effective absorption of VFA are critical physiological transitions for the neonatal calf. It is established that VFAs are the primary stimulant required for inducing rumen tissue development [39]. A massive community of microorganisms, bacteria, and protozoa ferment the plant material resulting in the production of VFAs, methane, and carbon dioxide. 

Interestingly, as discussed earlier, these same metabolites potentially regulate various physiological functions of the rumen [40,41]. VFAs are absorbed through the rumen epithelium and metabolized or transported directly into the bloodstream in the mature ruminant. In the ruminant, the rumen epithelium is the principal producer of ketone bodies. Acetoacetate, and β-hydroxybutyrate, mainly produced from the butyrate carbon absorbed from the rumen. These ketone bodies, along with acetate and propionate, are released into the blood and further metabolized by the liver to support the whole animal’s productive function. Ultimately, butyrate carbon captured in ketones, propionate and acetate serves as the primary sources of energy and carbon for the productive ruminant [41]. In mature rumen epithelium, butyrate serves as a preferred energy source for the rumen epithelial cells, promoting water and sodium absorption balance. Ultimately, metabolism may maintain blood pH by not releasing butyric acid directly into the blood [4,42]. Our recent report provided robust verification that butyrate induces specific epigenomic landscape changes and chromatin states, resulting in gene expression changes influencing rumen differentiation/development [35,43]. Butyrate stimulates the process resulting in functional (physical and metabolic) development of ruminal epithelium [44]. 

Confirming our earlier findings [15,45], and a study of [46], *PPARA* was upregulated and activated after weaning in the current study. It has been well documented that PPARα binds and is activated by numerous fatty acids and fatty acid-derived compounds [29,32]. The *PPARA* gene product is a ligand-activated transcription factor involved in regulating various processes, ranging from inflammation and immunity to nutrient metabolism and energy homeostasis [46]. PPARα governs biological processes by altering the expression of a large number of target genes. Accordingly, the specific role of PPARα is directly related to the biological function of its target genes [46]. Likewise, the PPAR isotype, PPARD, was upregulated and activated after weaning. Both PPARA and PPARD have been suggested to play a pivotal role in controlling biological processes driving fatty acid metabolism and crucial in regulating biological processes driving ruminal epithelial cell development [46]. Our findings in rumen tissues from concentrate-fed weaned calves are consistent with the increases in PPARD expression observed in the 10-week-old rumen from weaning age calves fed an enriched milk replacer compared to higher fat content milk replacer [39]. This may result from the decrease in circulating glucose and a move to cellular use of fatty acids to support energy metabolism and sustenance differentiation. Regardless, the expression changes of upstream regulators and transcription factors provide further evidence supporting the contention [35].

Another upstream regulator, PPARGC1A, exhibits a high activation post-weaning with a greater activation Z-score in the hay groups than those fed with grain. PPARGC1A, the protein (PPARG Coactivator 1 alpha, PGC-1 alpha) encoded by this gene, is a transcriptional coactivator that regulates the genes involved in energy metabolism. This protein interacts with PPAR gamma, which permits the interaction with multiple transcription factors [32]. Further, PPARGC1A interacts with and regulates cAMP response element-binding protein (CREB) and nuclear respiratory factors (NRFs). It provides a direct link between external physiological stimuli and the regulation of mitochondrial biogenesis. Additionally, PPARGC1A can regulate essential mitochondrial genes that contribute to the program of adaptive thermogenesis [47]. PPARGC1A is activated by signals that control energy and nutrient homeostasis [48], plays an essential role in metabolic reprogramming in response to dietary availability through coordination of the expression of a wide array of genes involved in glucose and fatty acid metabolism [25]. 

The e1F2 signaling pathway showed significantly upregulated activation at the late stage (10 weeks of age) of weaning. eIF2 is an essential factor for protein synthesis that forms a ternary complex (TC) with GTP and the initiator Met-tRNA. After its formation, the TC binds the 40S ribosomal subunit to form the 43S preinitiation complex. eIF2 plays a central role in maintaining a rate-limiting step in mRNA translation [49]. The e1F2 signaling pathway changes indicated that genetic activities during the weaning transition were at the transcription and the translation (protein synthesis) levels. The GO-term enrichment analysis results also reflect the process that gives rise to the functional (physical and metabolic) development of ruminal epithelium, which shows that the initiation, translation, and elongation of the protein translation are activated. Given the sizeable structural tissue changes and enzymes needed for metabolism, it is not surprising that protein synthesis is necessary to support the maturation of the tissue. Moreover, at ten weeks, the rumen microbiota is likely well established, and the tissue’s cornification is increasing to serve as a protective border.

Reduced activities in upstream regulators such as TGFB1, RICTOR, CLPP, MAP4K4, KDM5A, CCR2, and SRF were observed, corresponding to known or expected developmental changes ruminal tissue during weaning. For example, previous microarray analyses of tissues from the same study suggested a role of TGFB1 in rumen epithelial cell proliferation and differentiation [32]. Specifically, it was demonstrated in that study that *TGFB1* mRNA expression increased with rumen development during weaning. Likewise, a microarray study by Kim et al. [50] identified TGFB1 as an activated transcription factor in rumen epithelial biopsies obtained from dairy calves during the weaning transition. In the present analysis, as well as the study of the rumen primary epithelial cells treated with butyrate [35], we identified TGFB1 as a deactivated transcription factor based on RNA sequencing profiling and expression levels of its downstream targets. The reasons for the discrepancies are unclear. Possible explanations include a complete evaluation and a more sensitive analysis of changes in expression of downstream targets using RNA sequencing compared to microarray analysis, as well as a disconnect between transcript abundance of a transcription factor and the encoded protein’s effects on downstream gene targets. Some deactivated functions may reflect changes in metabolism occurring as the tissue begins to use VFA, such as fatty acid, fatty acid synthesis, and lipid concentration. Moreover, TGFB1 is an inducer of apoptosis and an essential negative regulator of proliferation [34]. Therefore, it is more conceivable that TGFB1 activity was downregulated during rumen development and growth. 

Of interest, KDM5A, a histone demethylase, was deactivated and played an essential role in blocking p53 signaling to reduce the activity of genes involved in translation initiation [51]. It also regulates the activity of Hox genes, which are critical for intestinal development and maturation [52] and functions in the repression of Notch signaling affecting tissue development, cell proliferation, and cell differentiation [53]. RICTOR, also detected as deactivated by TF analysis, is a negative regulator of cell growth and stem cell differentiation that functions through E-cadherin [54]. Its activity is regulated by nutrients [55] and multiple growth factors that can impact cell proliferation, survival, and tissue vascularization [56]. Likewise, deactivation of SRF may reflect changes in rumen epithelium related to the development of rumen epithelial layers and barrier function based on its critical functions in developing the skin epithelium in mice and connections between epithelial cells [57]. The role of these TF in rumen development has not been described in the scientific literature and warrant further study.

Integrative analysis of transcriptome reveals the stimulated biosynthesis of purine nucleotide, purine ribonucleotide, and nucleoside triphosphate as the combined effects of upstream regulators and downstream genes in the weaning group. Nucleic acids are important intracellular signaling molecules and coenzymes, the single most important means of coupling endergonic to exergonic reactions. Nucleic acids also store genetic information in DNA and RNA, chains of nucleotides made through DNA replication and transcription processes. Nucleoside triphosphates also serve as a source of energy for cellular reactions. Given their importance in the cell, the synthesis and degradation of nucleoside triphosphates are under tight control [58]. Moreover, purine metabolism pathways have been linked to butyrate’s biological effects in the bovine cell line experiment [59] and the epithelial tissue of cattle in lactation [60]. The stimulated biosynthesis of purine nucleotide, purine ribonucleotide, and nucleoside triphosphate is consistent with the necessity of rumen epithelial development and maturation during weaning. 

Our data indicated that MR+ G groups had delayed response in transcriptomic reprogramming during the transition, likely reflecting the continued reliance on post-ruminal absorption of energy as grains are acid hydrolyzed during the development of the microbiota in the rumen. This was reflected in the lower number of genes differentially expressed compared to the hay-fed groups. In mature dairy cows feeding of high grain diets results in lowered pH and ruminal dysbiosis, characterized by changes in absorption dynamics of VFAs across the reticulorumen wall, epithelial function, and the epithelial bacteria community structure. Thus, the regulatory pathways affecting altered rumen epithelial gene expression during the weaning process may be linked to well-documented ruminal acidosis symptoms, such as epithelial permeability, inflammation, and proliferation [41,61]. Considering the MR+G groups had delayed response during the weaning transition, a common pathway could play.

## 5. Conclusions

Shifts in gene expression have consisted of transcriptomic reprogramming of the rumen’s epithelial lining to promote and enable the developmental processes and support the maintenance of a functional mature organ. The conversion from reliance on glucose from milk to the use of microbial-fermentation end-products requires a complex transcriptional reprogramming of rumen epithelial tissue, and this reprogramming is driven by characterizing nutrient-gene interactions.

## Figures and Tables

**Table 1:** Statistics of RNA sequencing.

RNA-seq Statistics	Mean ± STD
Average Reads per sample	44,888,781 ± 5,226,700
Mapped pairs %	91.0 ± 2.4
Mapped to gene %	83.98 ± 0.53
Mapped to Intergenic %	16.02 ± 0.53
Forward % of reads mapped	50.37 ± 0.13
Reverse % of reads mapped	49.63 ± 0.12

STD: Standard Deviation.

## Data Availability

All RNA sequencing data were submitted to NCBI, SRA database (SUB3040669, BioProject ID: PRJNA658627, all data were published and accessible after Nov. 30, 2020).

## Supplementary Materials

The following are available online at https://www.mdpi.com/article/10.3390/ani11102870/s1, Table S1: PPARGC1A-target genes.
